# beta1-integrin mediates myelin-associated glycoprotein signaling in neuronal growth cones

**DOI:** 10.1186/1756-6606-1-10

**Published:** 2008-10-15

**Authors:** Eyleen LK Goh, Ju Kim Young, Kenichiro Kuwako, Marc Tessier-Lavigne, Zhigang He, John W Griffin, Guo-li Ming

**Affiliations:** 1 Institute for Cell Engineering, The Johns Hopkins University School of Medicine, MD 21205, USA; 2 Department of Neurology, The Johns Hopkins University School of Medicine, MD 21205, USA; 3 Division of Neuroscience, Children's Hospital, Boston, MA 02115, USA; 4 Division of Research, Genentech, 1 DNA Way, South San Francisco, CA 94080, USA; 5 The Solomon H. Snyder Department of Neuroscience, The Johns Hopkins University School of Medicine, MD 21205, USA

## Abstract

Several myelin-associated factors that inhibit axon growth of mature neurons, including Nogo66, myelin-associated glycoprotein (MAG) and oligodendrocyte myelin glycoprotein (OMgp), can associate with a common GPI-linked protein Nogo-66 receptor (NgR). Accumulating evidence suggests that myelin inhibitors also signal through unknown NgR-independent mechanisms. Here we show that MAG, a RGD tri-peptide containing protein, forms a complex with β1-integrin to mediate axonal growth cone turning responses of several neuronal types. Mutations that alter the RGD motif in MAG or inhibition of β1-integrin function, but not removal of NgRs, abolish these MAG-dependent events. In contrast, OMgp-induced repulsion is not affected by inhibition of b1-integrin function. We further show that MAG stimulates tyrosine phosphorylation of focal adhesion kinase (FAK), which in turn is required for MAG-induced growth cone turning. These studies identify β1-integrin as a specific mediator for MAG in growth cone turning responses, acting through FAK activation.

## Background

Myelin-associated glycoprotein (MAG), a component of myelin in the central and peripheral nervous system, promotes neurite outgrowth during the embryonic development, but inhibits axonal regeneration in the adult nervous system [1-9]. Following damage to the adult CNS, disruption of the myelin sheath leads to the release in abundance of a soluble fragment containing the MAG extracellular domain, which possesses potent inhibitory activity for neurite outgrowth [10]. A receptor complex consisting of NgR, p75/TROY and Lingo-1 has been shown to mediate the inhibitory activities of three major myelin-associated inhibitors: MAG, Nogo66 (an extracellular domain of NogoA) and OMgp [11-19]. While certain classes of neurons from p75 knockout mice exhibit reduced responses to myelin inhibitors, several types of neurons lacking NgRs are still inhibited by these factors [20-23]. In particular, a recent study using NgR germ-line knockout mice and short-hairpin RNA (shRNA) interference suggests that NgR is only partially involved in the acute growth cone collapse induced by MAG and OMgp, but may not be required for the long-term growth inhibitory actions of these two factor [22]. Thus, it is likely that an additional signaling mechanism is critical for transducing the signaling of MAG and possibly other myelin-associated inhibitors.

Integrins, consisting of α and β chains, are heterodimeric receptors for components of the extracellular matrix and for specific ligands [24]. Extensive studies have shown that integrins are important for cytoskeleton dynamics, cell adhesion and migration [25]. Emerging evidence also suggests that integrins regulate neurite extension, axonal guidance and neuronal migration through direct or indirect mechanisms [26]. Many downstream signaling of guidance cues and integrins converges onto common pathways that regulate cytoskeleton rearrangement, thus integrins and guidance cues could also modulate effects of each other [27-30]. In addition, exogenous laminin as a substrate impedes MAG and myelin inhibitory activity on neurite initiation and outgrowth [31,32]. These results suggest the existence of competitive crosstalk between integrin ligands and inhibitory factors associated with myelin and glia scar.

Here we demonstrated that β1-integrin acts as a receptor for MAG to mediate growth cone responses independent of NgRs in mammalian neurons. Our study identifies a novel signaling mechanism for MAG and may have significant implications for therapeutic modulation of MAG functions in the adult nervous system.

## Results

### MAG interacts with β1-integrin

**Figure 1:**
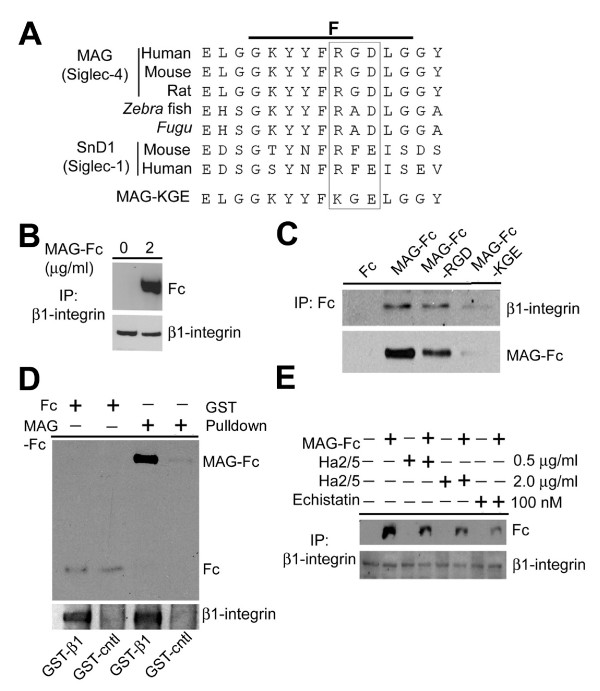
**Association between MAG and β1-integrin in primary hippocampal neurons**. A. Sequence alignment of the RGD motif in the F-strand of MAG (Siglec-4) and SnD1 (Siglec-1) from different species. B-E. Association between MAG and β1-integrin. Primary hippocampal cultures were treated with wild-type MAG-Fc (RGD), mutant MAG-Fc (KGE), or native Fc fragment, in the presence or absence of echistatin (100 nM) or Ha2/5 (0.5 or 2.0 μg/ml). Cell lysates were immunoprecipitated with antibodies raised against β1-integrin and subjected to immunobloting for human Fc fragment, or vice versa (B, C, E). In GST pull-down experiments (D), purified GST-β1-integrin (extracellular domain) or GST control was incubated with MAG-Fc or native Fc fragment, and GST pull-down was subjected to western blot analysis and immunobloting for the Fc fragment.

Human and rodent MAG (also called Siglec-4) contain the RGD tri-peptide (Fig. 1A), a characteristic binding motif recognized by integrin receptors containing β1 or β3 subunits [33,34]. Crystal structure analysis and modeling [35,36] suggest that the RGD motif in MAG (located within the F-strand, Fig. 1A) is not hidden from the protein surface as previously thought [37,38]. To determine whether β1-integrin interacts with MAG, we treated cultured primary hippocampal neurons with recombinant MAG consisting of the MAG extracellular domain fused to human Fc, a fusion protein previously shown to potently regulate neurite outgrowth when present uniformly and induce growth cone turning responses when applied locally [2,12,13,39-41]. MAG and β1-integrin were co-immunoprecipitated with antibodies directed against either β1-integrin or human Fc fragment (Fig. 1B, C), suggesting that these two proteins interact with each other. In contrast, native human Fc fragment and β1-integrin were not co-immunoprecipitated under the same condition (Fig. 1C). To further examine whether MAG directly interacts with β1-integrin, we purified recombinant protein of GST fused to the extracellular domain of β1-integrin. Pull-down experiments showed that GST-β1-integrin directly binds MAG-Fc, but not the native Fc fragment, in a cell free environment (Fig. 1D).

We next examined the requirement of the RGD motif in MAG for its association with β1-integrin. Biochemical analysis showed that the association between MAG and β1-integrin was attenuated by the disintegrin echistatin, a viper-venom-derived RGD peptide that specifically inhibitsβ1 and β3 containing integrins [42], and by Ha2/5, a specific β1-integrin function blocking antibody [43](Fig. 1E). We also constructed a mutant form of MAG (Fig. 1A), in which the RGD motif was mutated to KGE (MAG-KGE) and is not recognized by integrins [44]. Under the same experimental condition, purified MAG-KGE was unable to interact with β1-integrin whereas purified MAG-RGD (wild-type) could (Fig. 1C). Taken together, these results demonstrated that the association between MAG and β1-integrin is direct and occurs via a classical mode of integrin-ligand interaction[33,34].

### β1-integrin function is required for MAG-induced growth cone response

**Figure 2:**
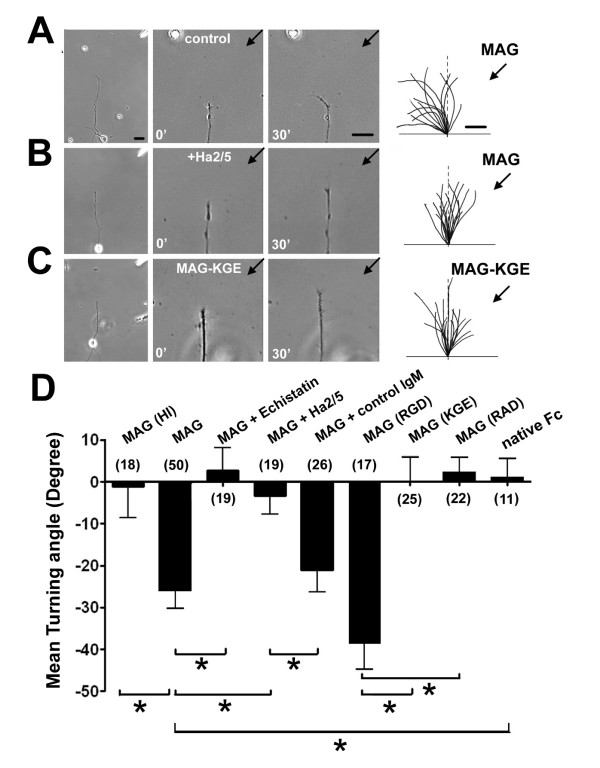
**β1-integrin function is essential for MAG-induced axonal growth cone repulsion of hippocampal neurons**. A-C, Growth cone turning in a gradient of MAG (150 μg/ml in the pipette). Sample images show the axons of P5 rat hippocampal neurons in the gradient for 30' on the left and axonal growth cones at the onset (0') and at the end (30') of the turning assay at a higher magnification. Scale bar: 20 μm. Right traces show sample trajectories of axons during the turning assay from 15 randomly selected neurons. Scale bar: 5 μm. D. Summary of growth cone turning angles under different conditions. Similar as in (A-C), growth cones were subjected a gradient of MAG-Fc, heat-inactivated (HI) MAG, wild-type (RGD) and mutant forms (KGE, RAD) of MAG-Fc, or native human Fc fragment. Pharmacological reagents were preincubated for 30 min and present throughout the turning assay with the following concentrations: echistatin (100 nM); Ha2/5 (1 μg/ml); Control IgM (1 μg/ml). Data represent mean ± s.e.m. Numbers associated with bars indicate the number of growth cones analyzed under each condition. "*" indicates significant difference (*p *< 0.01, ANOVA).

To examine the functional role of β1-integrin in transducing MAG signaling in neurons, we performed growth cone turning assays using rat hippocampal neurons [41,45] [See Materials and methods]. Consistent with earlier findings of repulsive growth cone responses of spinal neurons to MAG gradients [2,12,13,39-41], axonal growth cones of postnatal day 5 (P5) hippocampal neurons also exhibited repulsive responses in a microscopic gradient of recombinant MAG (150 μg/ml in the pipette; Fig. 2A). However, the growth cones showed no bias in the direction of axonal extension with heat-inactivated MAG-Fc (MAG-HI) or native Fc fragment (Fig. 2D). The repulsive responses were completely abolished in the presence of 100 nM echistatin or 1.0 μg/ml Ha2/5 (Fig. 2B, D), but not by the control IgM (Fig. 2D). Significant repulsive responses remained in the presence of a specific β3-integrin function-blocking antibody [see Additional file 1], suggesting the specific involvement of β1-integrin in mediating MAG induced growth cone response. Indeed, neurons transfected with specific shRNAs against β1-integrin [see Additional file 1], but not control shRNA, abolished growth cone responses to the MAG gradient [see Additional file 1]. Additionally, we generated two MAG mutants lacking an intact RGD motif: MAG-KGE or MAG-RAD. Gradients of mutant MAG proteins failed to induce significant growth cone response of these neurons (Fig. 2C &2D). Taken together, these results demonstrated that β1-integrin function is required for MAG-induced axonal growth cone repulsive response of postnatal hippocampal neurons.

**Figure 3:**
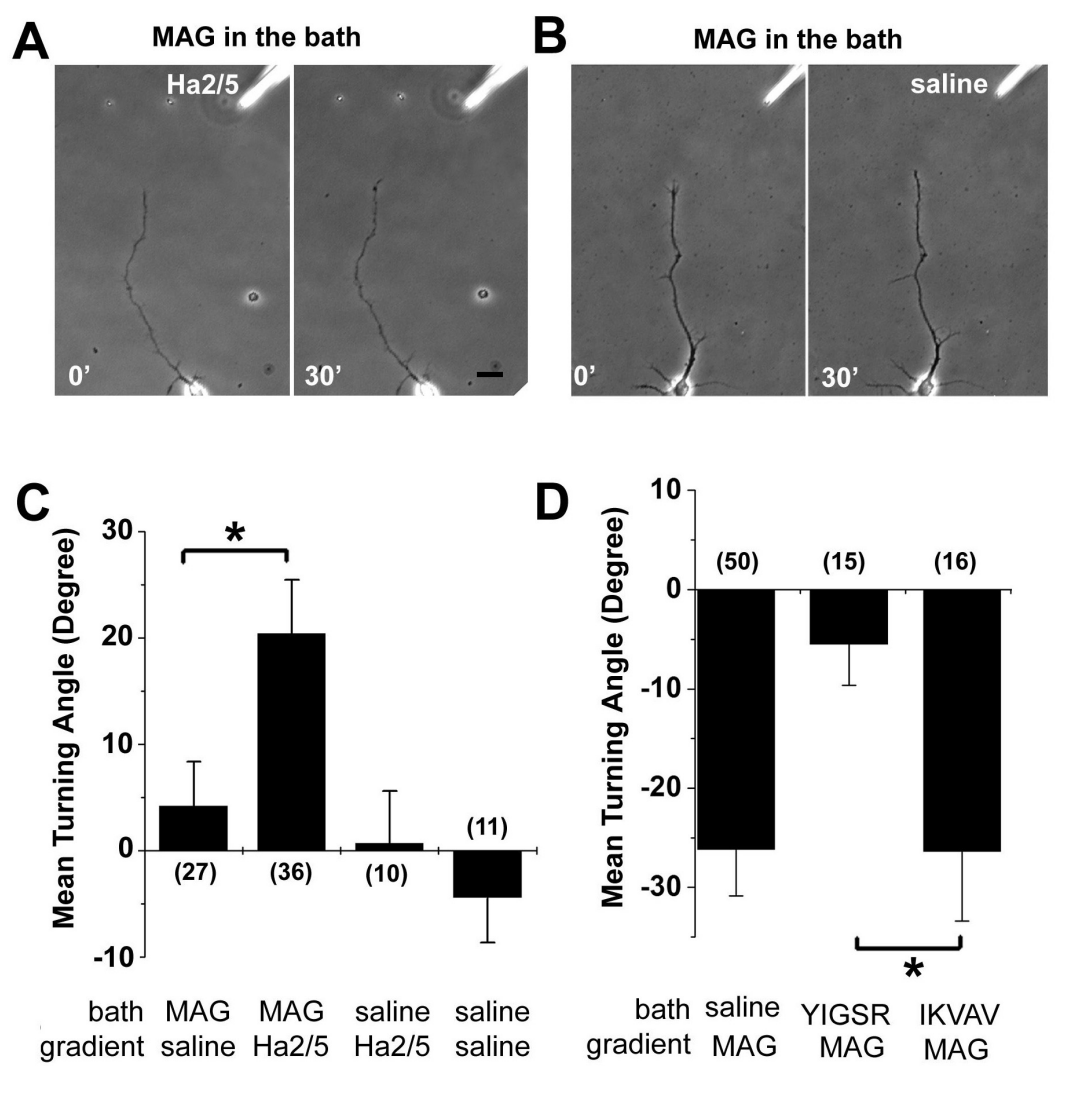
**β1-integrin plays an instructive role in MAG-induced growth cone turning**. A-C. Growth cone turning in the presence of uniform activation by MAG. Shown are sample images of growth cone turning of P5 rat hippocampal neuron axons in a gradient of Ha2/5 (0.5 mg/ml in the pipette, A) or saline (B) with uniform presence of MAG (150 ng/ml) in the bath. Scale bar: 20 μm. Also shown is the summary of growth cone turning angles under different conditions, C). Values represent mean ± s.e.m. Numbers associated with the bar graph indicate the number of growth cones analyzed. "*" indicates significant difference (*p *< 0.01, ANOVA). D. MAG-induced growth cone turning in the present of uniform activation of β1-integrin. Same as in (C), except that neurons were subjected to a MAG gradient (150 μg/ml in the pipette) with uniform presence of the β1-intergin activating peptide YIGSR (10 μg/ml), or the control peptide LKVAV (10 μg/ml), respectively.

To further characterize whether β1-integrin plays a permissive or an instructive role in MAG signaling, hippocampal neurons were uniformly activated by MAG in the bath (150 ng/ml) and exposed to a gradient of Ha2/5 (0.5 mg/ml in the pipette) to generate a reverse gradient of β1-integrin activation within the growth cone (Fig. 3A). Interestingly, neuronal growth cones exhibited significant attractive responses under this condition (Fig. 3A, C). In contrast, a control gradient of saline in the presence of uniform MAG (Fig. 3B), or a gradient of Ha2/5 in the absence of MAG, produced no significant growth cone turning responses (Fig. 3C). These results further demonstrated an essential role of β1-integrin in MAG-induced growth cone responses of hippocampal neurons. In another set of experiments, hippocampal neurons were subjected to the MAG gradient in the presence of bath application of a peptide consisting of five amino acid YIGSR, which has been previously shown to bind and activate β1-integrin, to saturate the β1-integrin signaling [46]. The repulsion to MAG was abolished by this uniform application of YIGSR, but not by a control peptide IKVAV (Fig. 3D). Thus, β1-integrin signaling appears to play an instructive rather than a permissive role in MAG-induced growth cone responses.

**Figure 4:**
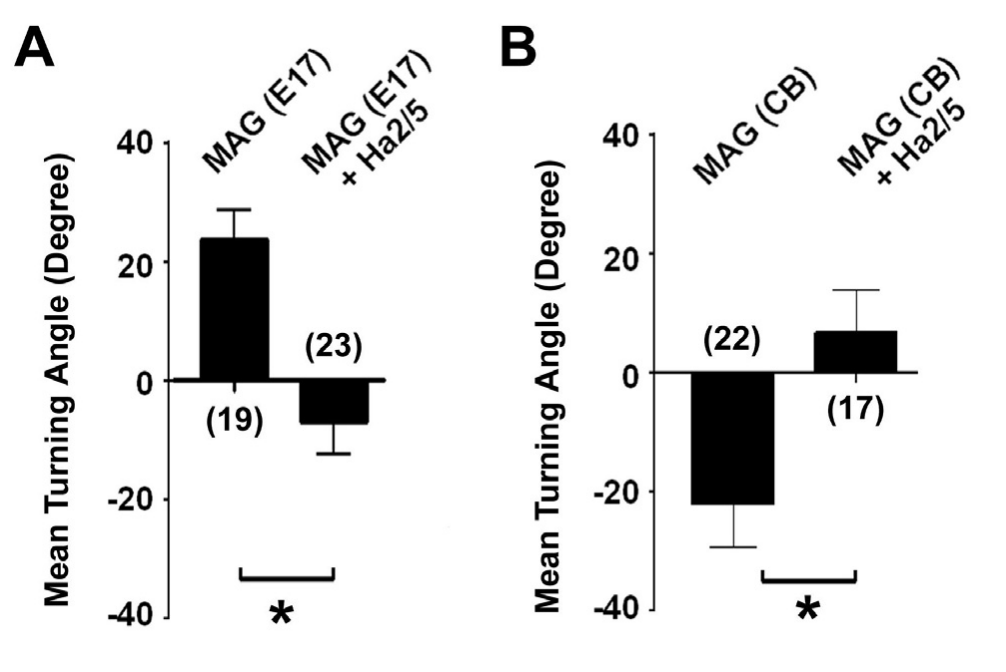
**β1-integrin mediates MAG-induced turning responses of embryonic hippocampal neurons and postnatal cerebellar neurons**. Shown is the summary of turning angles for axonal growth cones of rat E17 hippocampal neurons and P5 cerebellar neurons (CB) in a gradient of MAG (150 μg/ml in the pipette), with or without the presence of Ha2/5 (1.0 μg/ml). Values represent mean ± s.e.m. Numbers associated with the bar graph indicate the number of growth cones analyzed. "*" indicates significant difference (*p *< 0.01, ANOVA).

It is known that MAG exhibits differential effects on neurons at different developmental stages and, in particular, promotes neurite outgrowth of embryonic neurons [1,3,6,47]. We therefore tested whether β1-integrin also mediates growth cone responses of embryonic neurons to MAG. Interestingly, axonal growth cones of E17 rat hippocampal neurons exhibited significant attractive responses in the same MAG gradient, consistent with the growth promoting role of MAG on young neurons (Fig. 4A). Such MAG-induced response was also abolished in the presence of Ha2/5 (1.0 μg/ml; Fig. 4A). Neuronal responses to MAG are modulated by cAMP/PKA signaling [41,48]. Indeed, MAG-induced attractive responses of E17 neurons was converted to repulsive responses in the present of a PKA inhibitor Rp-cAMPS (20 μM), while MAG-induced repulsive responses of P5 hippocampal neurons was converted to attractive responses in the presence of a PKA activator Sp-cAMPS (20 μM; [see Additional file 2]). Importantly, all these MAG-induced growth cone responses were abolished in the presence of Ha2/5 [see Additional file 2]. Thus, β1-integrin mediates MAG-induced growth cone responses of hippocampal neurons at different developmental stages and under different cellular status.

To determine whether the function of MAG-integrin interactions are limited to hippocampal neurons, we examined growth cone responses of postnatal rat cerebellar granule cells [45]. Axonal growth cones of these neurons exhibited significant repulsive responses in the MAG gradient (150 μg/ml in the pipette; Fig. 4B). Importantly, MAG-induced repulsion of these neurons was also abolished in the presence of Ha2/5 (1.0 μg/ml; Fig. 4B). These results show that β1-integrin function is required for MAG-induced growth cone responses in different types of mammalian CNS neurons.

### β1-integrin function is not required for OMgp-induced growth cone turning

**Figure 5:**
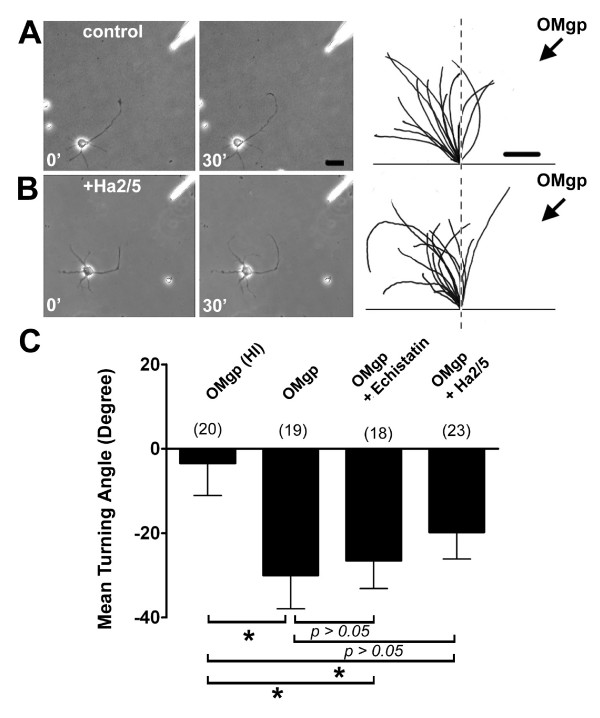
**OMgp-induced growth cone repulsion does not require β1-integrin function**. A. Growth cone turning of rat hippocampal neurons in a gradient of OMgp. Sample images show an axon of a P5 hippocampal neuron in an OMgp gradient (5 μg/ml in the pipette) at the onset (0') and at the end (30') of the turning assay. Scale bar: 20 μm. Right traces show sample trajectories of axons during the turning assay from 15 randomly selected neurons. Scale bar: 5 μm. B. Growth cone turning in a gradient of OMgp in the presence of a blocking antibody to β1-integrin. Similar as in (A), except for the presence of Ha2/5 (1.0 μg/ml). C. Summary of growth cone turning angles under different conditions. Echistatin (100 nM) or Ha2/5 (1 μg/ml) was preincubated for 30 min and present throughout the turning assay. Values represent mean ± s.e.m. Numbers associated with the bar graph indicate the number of growth cones analyzed. "*" indicates significant difference from the heat-inactivated (HI) OMgp (*p *< 0.01, ANOVA).

Three major myelin-associated inhibitory factors, Nogo-66, MAG and OMgp, are known to bind to the common NgR protein and may utilize the same signal transduction pathway to regulate axonal behaviours [11-14]. Therefore, we next sought to determine whether β1-integrin also mediates growth cone responses to other myelin-associated inhibitors. Axonal growth cones of rat P5 hippocampal neurons exhibited significant repulsive turning responses to a gradient of recombinant OMgp (5 μg/ml in the pipette), but not to Nogo-66 (data not shown) or to heat-inactivated OMgp (Fig. 5A, C). In contrast to our observations for MAG, axonal growth cones still exhibited significant repulsive response to OMgp in the presence of either echistatin or Ha2/5 (Fig. 5B, C). Thus, β1-integrin appears to specifically mediate axonal growth cone responses induced by MAG, but not by OMgp.

### β1-integrin mediates MAG-induced growth cone turning independent of NgR

**Figure 6:**
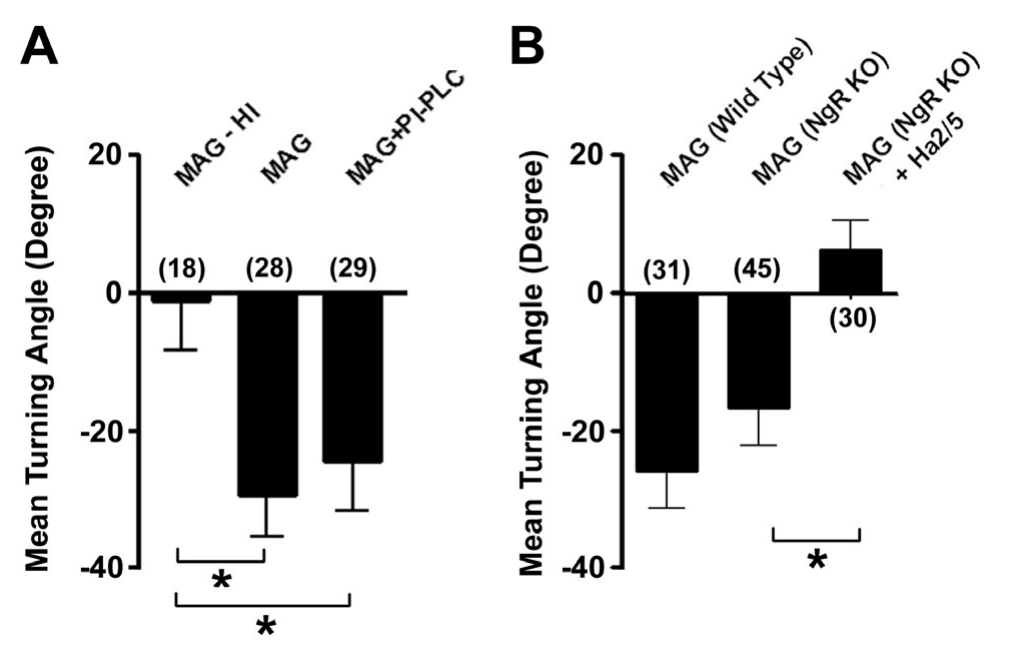
**NgRs are dispensable for MAG-induced growth cone repulsion of hippocampal neurons**. A. MAG-induced growth cone turning after the PI-PLC treatment. Primary hippocampal neurons were pre-treated with PI-PLC (1 unit/ml) for 30 min at 37°C and then growth cones were examined in a gradient of MAG. Shown is the summary of turning angles of axons with or without the PI-PLC treatment. B. MAG-induced growth cone turning of hippocampal neurons from NgR knockout mice and WT littermates. Shown is the summary of turning angles for axonal growth cones of P5 mouse hippocampal neurons derived from wild-type or NgR knockout (NgR KO) mice in a gradient of MAG (150 μg/ml in the pipette) with or without Ha2/5 (1.0 μg/ml) in the bath. Values represent mean ± s.e.m. Numbers associated with the bar graph indicate the number of growth cones analyzed. "*" indicates significant difference (*p *< 0.01, ANOVA).

To address whether β1-integrin provides an independent pathway to mediate MAG signaling or acts as a co-receptor along with NgR/p75/TROY/Lingo-1, we examined growth cone responses following the removal of GPI-linked proteins, including all NgRs, from the neuronal cell surface [13,14]. Primary hippocampal neurons were pre-treated with PI-PLC (1 unit/ml) for 30 min and then growth cones were examined in the MAG gradient with the continuous presence of PI-PLC. Under these conditions, axonal growth cones of P5 rat hippocampal neurons still exhibited significant repulsive turning responses to MAG (Fig. 6A). Biochemical analysis confirmed that the PI-PLC treatment was effective in removing NgR from these primary neurons [see Additional file 3], but the binding of MAG to β1-integrin was not affected [see Additional file 3]. These results are consistent with a number of previous findings that MAG retains its ability to induce RhoA activation [20] and inhibit neurite outgrowth[23] in postnatal cerebellar granule cells following the PI-PLC treatment, while mutant forms of MAG-Fc lacking an intact RGD domain (AGD or DGD) lose their inhibitory activities on axonal extension of cultured cerebellar granule cells [49].

To directly assess the specific role of NgR in MAG-induced growth cone responses, we examined neurons from NgR null mice [21]. Mouse hippocampal neurons lacking NgR still exhibited significant repulsive responses to the MAG gradient (Fig. 6B), suggesting that NgR is dispensible for MAG-induced growth cone repulsion. More importantly, MAG-induced repulsion of neurons lacking NgR was also abolished by Ha2/5 (Fig. 6B). In addition, we were unable to detect interactions between β1-integrin and any member of the known NgR signaling complex, including NgR, p75, TROY and Lingo-1, either in the presence or absence of MAG [see Additional file 4]. Taken together, these findings are consistent with the notion that β1-integrin mediates MAG-induced growth cone responses independent of the known NgR receptor complex.

### FAK mediates MAG-induced growth cone turning downstream of β1-integrin

**Figure 7:**
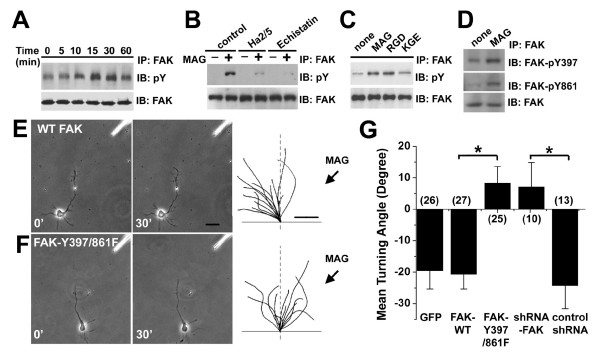
**MAG-induced tyrosine phosphorylation of FAK is required for growth cone repulsion to MAG**. A-C. MAG induces phosphorylation of FAK. Shown in (A) is the time course of FAK phosphorylation after MAG stimulation (2 μg/ml) of rat hippocampal neurons. Cell lysates were immunoprecipitated with anti-FAK antibodies and immunoblotted with the pY-20 antibody for phosphorylated tyrosine residues. Shown in (B) are experiments in the presence or absence of Ha2/5 (1.0 μg/ml) or echistatin (100 nM). Shown in (C) are experiments with the treatment of WT-MAG (RGD) or mutant MAG (KGE). D. MAG induces phosphorylation of FAK on residues Y397 and Y861. Cell lysates of hippocampal neurons after MAG stimulation were immunoprecipitated with anti-FAK antibodies and immunoblotted with tyrosine phosphorylation site-specific antibodies to FAK. E-G, Phosphorylation of FAK on residues Y397 and Y861 is required for MAG-induced growth cone repulsion. Hippocampal neurons were transfected with expression constructs for GFP, WT-FAK-GFP (E), FAK-Y397/861F-GFP (F), GFP and control shRNA, GFP and shRNAs against FAK. Growth cones of GFP^+ ^neurons were examined in a gradient of MAG (150 μg/ml in the pipette). Sample images and traces were shown similarly as in Fig. 2 (A-C). Scale bar: 20 μm for microscopic images and 5 μm for traces. Shown in (G) is the summary of growth cone turning angles. Values represent mean ± s.e.m. Numbers associated with the bar graph indicate the number of growth cones analyzed. "*" indicates significant difference from the control (neurons transfected to express GFP alone; *p *< 0.01, ANOVA).

How does β1-integrin signaling transduce MAG-induced growth cone responses? Focal adhesion kinase (FAK) is a major mediator of integrin-dependent signaling in many contexts, including cell migration and axon guidance [26,50,51]. Interestingly, treatment of hippocampal neurons with MAG (2 μg/ml) induced tyrosine phosphorylation of FAK in a time-dependent manner (Fig. 7A). Such MAG-induced tyrosine phosphorylation of FAK was abolished in the presence of echistatin (100 nM) or Ha2/5 (2.0 μg/ml; Fig. 7B). In addition, mutant MAG-KGE failed to trigger tyrosine phosphorylation of FAK (Fig. 7C) while removing GPI-linked proteins following the PI-PLC treatment did not affect MAG-induced phosphorylation of FAK in these neurons [see Additional file 5]. Thus, MAG induces tyrosine phosphorylation of FAK in an integrin-dependent manner.

We further examined specific tyrosine residues of FAK that are phosphorylated upon MAG stimulation in hippocampal neurons. As shown with site-specific phospho-tyrosine FAK antibodies, MAG induced a significant increase in the phosphorylation of FAK at tyrosine residues 397 and 861 (Fig. 7D). Similar β1-integrin-dependent phosphorylation of FAK these tyrosine residues were also found in embryonic cortical neurons treated with MAG (data not shown).

To determine the functional role of FAK and its tyrosine phosphorylation in MAG-induced growth cone turning, we transfected P5 rat hippocampal neurons with shRNA constructs to knockdown the expression of endogenous FAK [see Additional file 5]. Expression of shRNA-FAK, but not control shRNA, abolished MAG-induced repulsion (Fig. 7G). We also transfected neurons with expression constructs for either wild-type FAK (WT-FAK) or a mutant FAK (FAK-Y397/861F) that cannot be phosphorylated on tyrosine residues 397 and 861. Expression of mutant FAK-Y397/861F, but not WT-FAK, also abolished MAG-induced repulsion (Fig. 7E–G). Together, these findings demonstrated that MAG-induced phosphorylation of FAK is essential for growth cone turning responses to MAG.

## Discussion

We provided biochemical and functional evidence that β1-integrin acts as a direct receptor to mediate MAG-induced growth cone responses of mammalian CNS neurons from both embryonic and postnatal stages. We further showed that β1-integrin signaling mediates MAG effects through FAK phosphorylation and is independent of NgR. Taken together, these results demonstarted a common role of β1-integrin in mediating MAG signaling for diverse functions in different neuronal types.

Previous studies led to the finding that Nogo66, OMgp and MAG, three major inhibitors associated with myelin, all bind to NgR and appear to signal at axonal growth cones through a common receptor complex containing NgR, p75/TROY and Lingo-1 [11-19]. Two additional human homologs of NgR (NgR2 and NgR3) are found to be expressed in CNS neurons [52,53]. While neither binds to Nogo66 [54], NgR2 appears to bind to MAG [55]. Accumulating evidence suggests that inhibitors associated with myelin may signal independent of NgRs [20-22]. Our growth cone turning results using NgR null neurons and PI-PLC treatment are in agreement with these findings (Fig. 6). MAG has also been reported to inhibit neurite outgrowth through sialoglycoproteins [49,56] and gangliosides [23,57] in postnatal DRG neurons and cerebellar granule neurons. Our results with the RAD mutant that has an intact arginine residue to mediate the binding of MAG to sialic acids [49] (Fig. 2) but failed to induce growth cone responses suggest a specific requirement of β1-integrin in MAG signaling. Whether sialic acids of sialoglycoproteins and gangliosides serve as a co-receptor together with β1-integrin to mediate MAG signaling remains to be determined [58].

Integrin signaling has been shown to be critical for axon guidance and cell migration, either as a direct receptor or as a modulator of guidance signaling [26]. Laminins, when presented as substrates for integrins, are known to promote neurite outgrowth [59] and have been shown to override inhibitory activities of MAG and myelin-associated factors [31,32]. It is possible that, in addition to the growth promoting activity of laminin, competitions at the receptor levels by laminins and MAG may also contribute to the enhancement of neurite initiation and outgrowth [60,61]. Our results also support the notion that integrin signaling plays an instructive, rather than permissive role, in MAG-induced growth cone turning (Fig. 3). Activation of the integrin/FAK pathway is normally associated with enhanced nerve growth/growth cone attraction [26,62]. Interestingly, β1-integrin signaling is required for both MAG-induced repulsion and attraction of CNS neurons at different developmental stages and under different cellular status (Fig. 4; [see Additional file 2]). A recent study also showed that inhibition of neurite outgrowth by fibrinogen requires β3-integrin function [63]. Taken together, these findings suggest a bi-functional role of integrin/FAK signaling in regulating the dynamics of cytoskeletal proteins. Our results show that β1-integrin serves as a specific receptor for MAG, but not for OMgp. Consistent with the selective involvement of β1-integrin in mediating MAG effects, human and rodent MAG contain a RGD-tri-peptide motif characteristic of integrin binding proteins [33,34], whereas OMgp and Nogo do not. Interestingly, MAG homologs in *fugu *and *Zebrafish*, species with the capacity for axonal regeneration, do not contain an intact RGD motif (Fig. 1). The extent to which different receptors mediate distinct effects of MAG in various species remains to be determined. Our results further demonstrate that integrin/FAK signaling mediates MAG effects independent of the NgR receptor complex. These findings suggest that a diversity of signaling mechansims is likely to be employed to limit axon regeneration in the adult CNS. Given the general role of β1-integrin in mediating diverse functions of MAG in the adult central nervous system, our findings may have implications for novel strategies for therapeutic modulation of MAG functions in the adult nervous system.

Our results show that β1-integrin serves as a specific receptor for MAG, but not for OMgp. Consistent with the selective involvement of β1-integrin in mediating MAG effects, human and rodent MAG contain a RGD-tri-peptide motif characteristic of integrin binding proteins [33,34], whereas OMgp and Nogo do not. Interestingly, MAG homologs in *fugu *and *Zebrafish*, species with the capacity for axonal regeneration, do not contain an intact RGD motif (Fig. 1). The extent to which different receptors mediate distinct effects of MAG in various species remains to be determined. Our results further demonstrate that integrin/FAK signaling mediates MAG effects independent of the NgR receptor complex. These findings suggest that a diversity of signaling mechansims is likely to be employed to limit axon regeneration in the adult CNS. Given the general role of β1-integrin in mediating diverse functions of MAG in the adult central nervous system, our findings may have implications for novel strategies for therapeutic modulation of MAG functions in the adult nervous system.

## Methods

### Primary neuronal cultures

Hippocampal neurons were isolated from the hippocampi embryonic and postnatal rats, or wild-type and NgR knockout mice [21] as previously described [64]. Similarly, cerebellar neurons were isolated from P5 rat cerebellum [45]. Dissociated neurons were cultured on poly-L-lysine coated plates or coverslips without laminin as previously described [64]. For biochemical analysis, E18 neurons were treated with AraC to eliminate dividing astrocytes and used at 5 days after plating as previously described [64]. For growth cone turning assay, neurons were used between 2–3 days after plating. PI-PLC (1 or 2 units/ml)[14], Ha2/5 (1 μg/ml) or echistatin (100 nM) [42] were added 30 mins prior to and were present during the growth cone turning assay.

### Expression constructs and neuronal transfection

Mutation of MAG-Fc was generated by site directed mutagenesis and confirmed by DNA sequencing. Expression plasmids of wild-type MAG (RGD) or mutant forms of MAG (KGE, RAD) were transfected into 293 Ebna cells and proteins were collected from the media and affinity purified using protein A sepharose. MAG-Fc from R & D systems was also used. The pUEG vector was used to co-express GFP (under the control of the EF1α promoter) and a specific shRNA (under the control of the human U6 promoter in the same vector)[65,66]. Several shRNAs against different regions of β1-integrin or FAK, and control shRNA against DsRed [65] were generated. The following short-hairpin sequences were cloned into pUEG vector using a PCR SHAGing strategy [67]: shRNA-control: AGTTCCAGTACGGCTCCAA; shRNA-β1-integrin-3: TGCCTACTTCTGCACGATG; shRNA-FAK1: GCACGTGGCCTGCTATGGA; shRNA-FAK2: GCCTTAACAATGCGTCAGT; and shRNA-FAK3: TCCAGAAGACAGGCTACCG. To validate the specificity and efficiency of shRNAs, pUEG vectors with different shRNAs were transfected into 3T3 cells and cell lysates were prepared for western blot analysis of β1-integrin or FAK expression with specific antibodies, respectively.

Rat primary hippocampal neurons were transfected with the *Amaxa *transfection system following protocols from the manufacturer. Briefly, hippocampal neurons were isolated and 100 μl of nucleofector solution was added to resuspend the cell pellet. Different expression constructs (1–5 μg) for GFP, WT-FAK-GFP, FAK-Y397/861F-GFP[68], WT-Rho-GFP, DN-Rho-GFP, or pUEG vectors for shRNAs [65,66], were added to the cell suspension and the cell-DNA mix was then transferred to cuvettes for electroporation. The cells were cultured in DMEM with 10% fetal bovine serum for 24 hrs before changing to the serum-free neurobasal medium [64]. GFP^+ ^neurons were identified for the turning assay.

### Biochemistry

Neurons at 5 days after plating were treated with 2 units/ml PI-PLC, 100 nM Echistatin or 0.5–2.0 μg/ml Ha2/5, and then stimulated with 2.0 μg/ml MAG or 0.5 μg/ml OMgp for the indicated time periods. Cells were then lysed in immunoprecipitation buffer (1% Triton X-100; 150 mM NaCl; 10 mM Tris, pH 7.4; 1 mM EDTA; 1 mM EGTA; 1% Nonidet P-40; 0.2 mM Na_3_VO_4_; 1 μg/ml protease inhibitor cocktail; and 0.1 mM PMSF). Samples were immunoprecipitated with polyclonal antibody against FAK (Santa Cruz Biotechnology, Inc.), human Fc (Sigma) or β1-integrin (Chemicon), and then subjected to western blot analysis. The following antibodies were used: monoclonal antibody against tyrosine phosphorylated proteins (pY20, Transduction Laboratories; 1:1000), rabbit polyclonal antibodies against β1-integrin (1:1000), FAK (1:1000), FAK-pY397 (Biosource; 1:1000), FAK-pY861 (Biosource; 1:1000), or human Fc (1:1000). Blots were stripped and reblotted with the same antibodies used for their immunoprecipitation to ensure equal loading of the immunoprecipitated proteins.

For GST pull-down experiments, the extracellular domain (ECD) of β1-integrin was amplified from mouse brain cDNA and cloned into the GST-fusion expression vector (pGEX-4T-1; Amersham-Pharmacia Biotech) to express GST-β1 (ECD) fusion protein. The fusion protein was purified using glutathione beads according to the manufacturer's manual (Amersham-Pharmacia Biotech). Native Fc fragment (2 μg/ml) or MAG-Fc (2 μg/ml) was then added to the purified GST-β1 (ECD) overnight at 4°C. The samples were further processed according to the standard immunoprecipitation protocol as described.

For experiments testing potential interactions between β1-integrin and the NgR receptor complex, HEK293 cells were transfected with expression constructs for NgR, p75, TROY, or Lingo-1, respectively, as previously described [18]. Transfected cells were stimulated with MAG (5 μg/ml) or medium and then were immunoprecipitated with anti-β1-integrin antibodies and immunoblotted for respective components of the NgR receptor complex. Total cell lysates were also examined to show the expression of endogenous β1-integrin and proteins from transfection.

### Growth cone turning assay

Microscopic gradients of recombinant MAG (150 μg/ml in the pipette; 1.8 μM) and OMgp (5 μg/ml in the pipette; 0.1 μM) were produced as previously described to induce growth cone turning responses [40,41,45,69]. In some experiments, MAG (150 ng/ml) was added to the bath solution and microscopic gradients were produced with saline or Ha2/5 (0.5 μg/μl) in the pipette. As another control, a gradient of Ha2/5 (0.5 μg/μl in the pipette) was applied in the absence of MAG in the bath. Previous analysis [69,70] have shown that, under standard pulsing conditions, the average concentration of the factor at the growth cone at a distance of 100 μm from the pipette tip is about 10^3 ^fold lower than that in the pipette and the concentration gradient across the growth cone is about 5–10%. Axons were identified as the longest neurite in these cultures at stage 2–3 of hippocampal neurons as previously described [71]. Growth cone assays were carried out for 30 min at room temperature. The turning angle was defined by the angle between the original direction of neurite extension and a line connecting the position of the center of the growth cone at the onset and the end of the 30 min period. To assure accurate measurement of turning angles, only neurons with axonal extension > 5 μm over the 30 min period were included for analysis.

## Competing interests

The authors declare that they have no competing interests.

## Authors' contributions

ELKG carried out all biochemistry studies. JYK carried out the all growth cone turning assays. KK and ZH participated in the interaction studies between β1-integrin and NgR components. GLM conceived of the study. MTL, ZH, JWG and GLM participated in its design and coordination and helped to draft the manuscript. All authors read and approved the final manuscript.

## Supplementary Material

Additional file 1 **Requirement of β1-integrin function in MAG-induced growth cone turning of hippocampal neurons under different conditions**. **A**. Summary of turning angles for axonal growth cones of P5 rat hippocampal neurons expressing control shRNA or shRNA specific against β1-integrin, or in the presence of anti-β3-integrin antibody. **B**. Sample western blot for 3T3 cells transfected with vectors expressing control shRNA or specific shRNAs against FAK, and immunoblotted for FAK and β-actin. Click here for file

Additional file 2 **Summary of turning angles for embryonic and postnatal axonal growth cones of E17 embryonic and P5 postnatal hippocampal neurons in the presence of pharmacological PKA activator (Sp-cAMPS, 20 μM) or inhibitor (Rp-cAMPS, 20 μM), respectively**. Note the conversion of MAG-induced attraction of E17 neurons to repulsion by inhibition of PKA and conversion of MAG-induced repulsion of P5 neurons to attraction by activation of PKA. Importantly, all MAG-induced turning responses were abolished by the bath application of Ha2/5 (1.0 μg/ml). Values represent mean ± s.e.m. Numbers associated with the bar graph indicate the number of growth cones analyzed. "*" indicates significant difference (*p *< 0.01, ANOVA). Click here for file

Additional file 3 **Effects of the PI-PLC treatment on hippocampal neurons**. **A**. Effective removal of NgR from primary hippocampal neurons by the PI-PLC treatment. Primary hippocampal neurons were transfected with the empty vector or vector expressing NgR-FLAG. Cultures with or without the PI-PLC treatment (1 unit/ml for 30 min at 37°C) were subjected to western blot analysis using antibodies against the FLAG tag. The membrane was reblotted for GADPH to show similar loading. **B**. The PI-PLC treatment does not affect binding of MAG to β1-integrin in hippocampal neurons. Hippocampal neurons were treated with saline or PI-PLC (1 units/ml) for 30 min at 37°C, then stimulated with MAG (1 μg/ml) for 15 min. Cell lysates were immunoprecipitated with anti-β1 integrin antibodies and immunoblotted with anti-Fc antibodies. The membrane was reblotted for β1-integrin to show similar loading. Click here for file

Additional file 4 **Lack of interaction between β1-integrin and members of the known NgR receptor complex**. Lysates of HEK293 cells transfected with expression constructs for NgR, p75, TROY, or Lingo-1, with or without MAG treatment (5 μg/ml), were immunoprecipitated with anti-β1-integrin antibodies and immunoblotted for the respective components of the NgR receptor complex. Also shown are immunoblots for total cell lysates showing the expression of endogenous β1-integrin and transfected proteins, and reblots for Fc showing a strong association of MAG and β1-integrin in 293 cells. Click here for file

Additional file 5 **FAK activation independent of PI-PLC**. **A**. The PI-PLC treatment does not affect MAG-induced phosphorylation of FAK in hippocampal neurons. Hippocampal neurons were treated with saline or PI-PLC (1 units/ml) for 30 min at 37°C, then stimulated with MAG (1 μg/ml) for 15 min. Cell lysates were immunoprecipitated with anti-FAK antibodies and immunoblotted with pY-20 antibody for phosphorylated tyrosine residues. The membrane was reblotted for FAK to show similar loading. **B**. Knockdown of the expression of endogenous FAK by specific shRNAs. Sample western blot of 3T3 cells were transfected with vectors expressing control shRNA or specific shRNAs against FAK, and then immunoblotted for FAK and β-actin. Click here for file
