# Supplementary figures and images for: beta1-integrin mediates myelin-associated glycoprotein signaling in neuronal growth cones
Source: Mol Brain. 2008 Oct 15;1:10. doi: 10.1186/1756-6606-1-10 (PMC2576245; doi:10.1186/1756-6606-1-10)

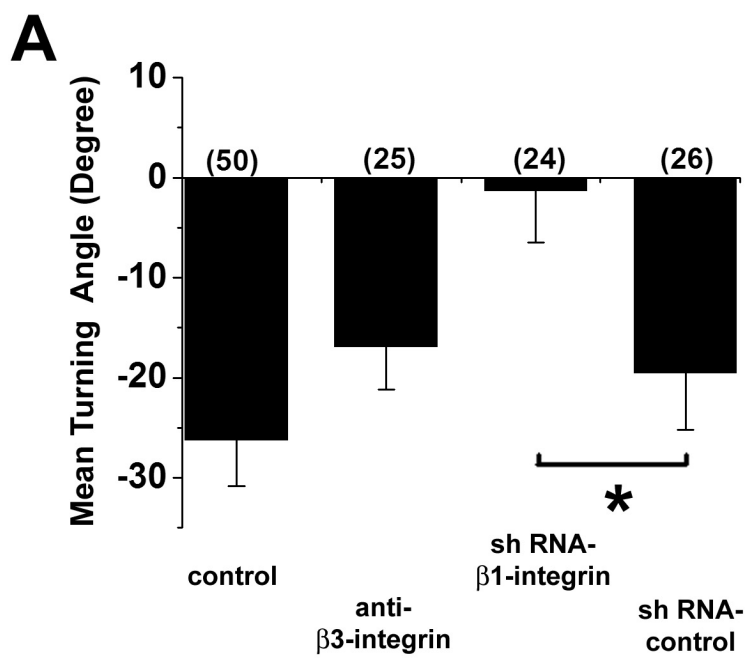

**B**

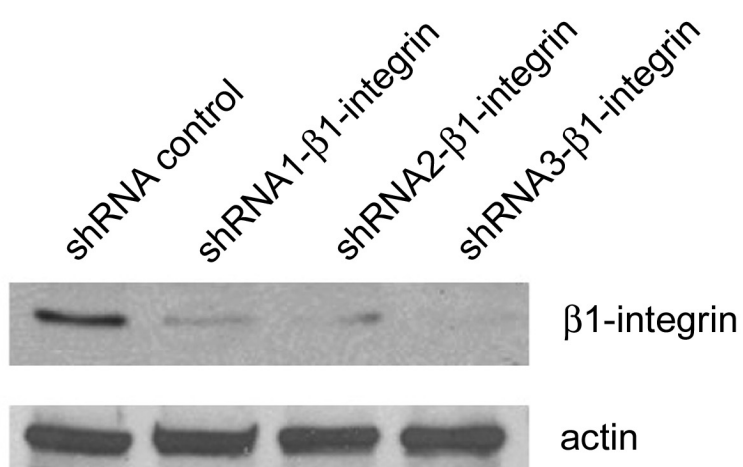

Supplement: Additional file 1 — Requirement of β1-integrin function in MAG-induced growth cone turning of hippocampal neurons under different conditions. A. Summary of turning angles for axonal growth cones of P5 rat hippocampal neurons expressing control shRNA or shRNA specific against β1-integrin, or in the presence of anti-β3-integrin antibody. B. Sample western blot for 3T3 cells transfected with vectors expressing control shRNA or specific shRNAs against FAK, and immunoblotted for FAK and β-actin. [file 1756-6606-1-10-S1.pdf]

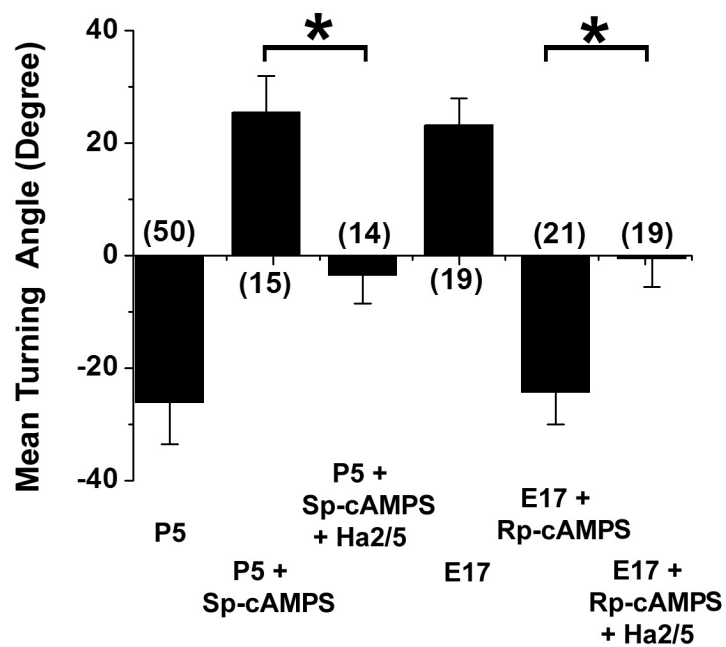

Supplement: Additional file 2 — Summary of turning angles for embryonic and postnatal axonal growth cones of E17 embryonic and P5 postnatal hippocampal neurons in the presence of pharmacological PKA activator (Sp-cAMPS, 20 μM) or inhibitor (Rp-cAMPS, 20 μM), respectively. Note the conversion of MAG-induced attraction of E17 neurons to repulsion by inhibition of PKA and conversion of MAG-induced repulsion of P5 neurons to attraction by activation of PKA. Importantly, all MAG-induced turning responses were abolished by the bath application of Ha2/5 (1.0 μg/ml). Values represent mean ± s.e.m. Numbers associated with the bar graph indicate the number of growth cones analyzed. "*" indicates significant difference (p < 0.01, ANOVA). [file 1756-6606-1-10-S2.pdf]

**A**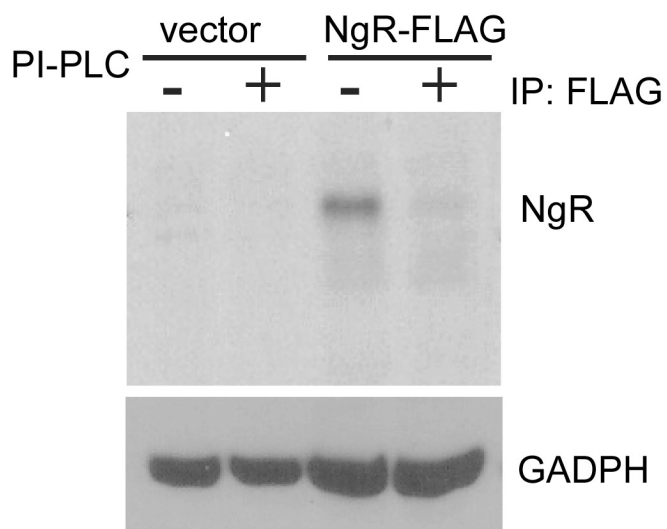**B**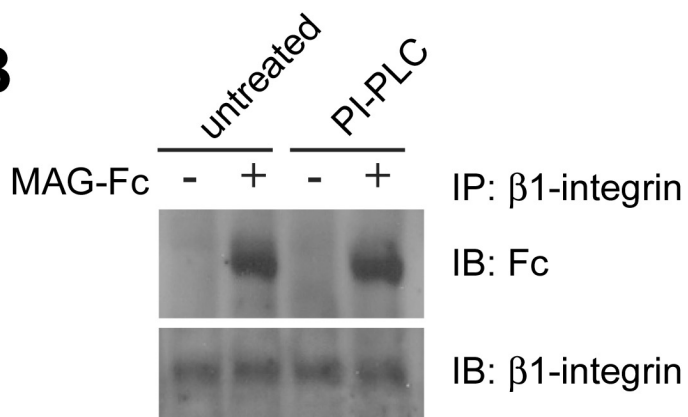

Supplement: Additional file 3 — Effects of the PI-PLC treatment on hippocampal neurons. A. Effective removal of NgR from primary hippocampal neurons by the PI-PLC treatment. Primary hippocampal neurons were transfected with the empty vector or vector expressing NgR-FLAG. Cultures with or without the PI-PLC treatment (1 unit/ml for 30 min at 37°C) were subjected to western blot analysis using antibodies against the FLAG tag. The membrane was reblotted for GADPH to show similar loading. B. The PI-PLC treatment does not affect binding of MAG to β1-integrin in hippocampal neurons. Hippocampal neurons were treated with saline or PI-PLC (1 units/ml) for 30 min at 37°C, then stimulated with MAG (1 μg/ml) for 15 min. Cell lysates were immunoprecipitated with anti-β1 integrin antibodies and immunoblotted with anti-Fc antibodies. The membrane was reblotted for β1-integrin to show similar loading. [file 1756-6606-1-10-S3.pdf]

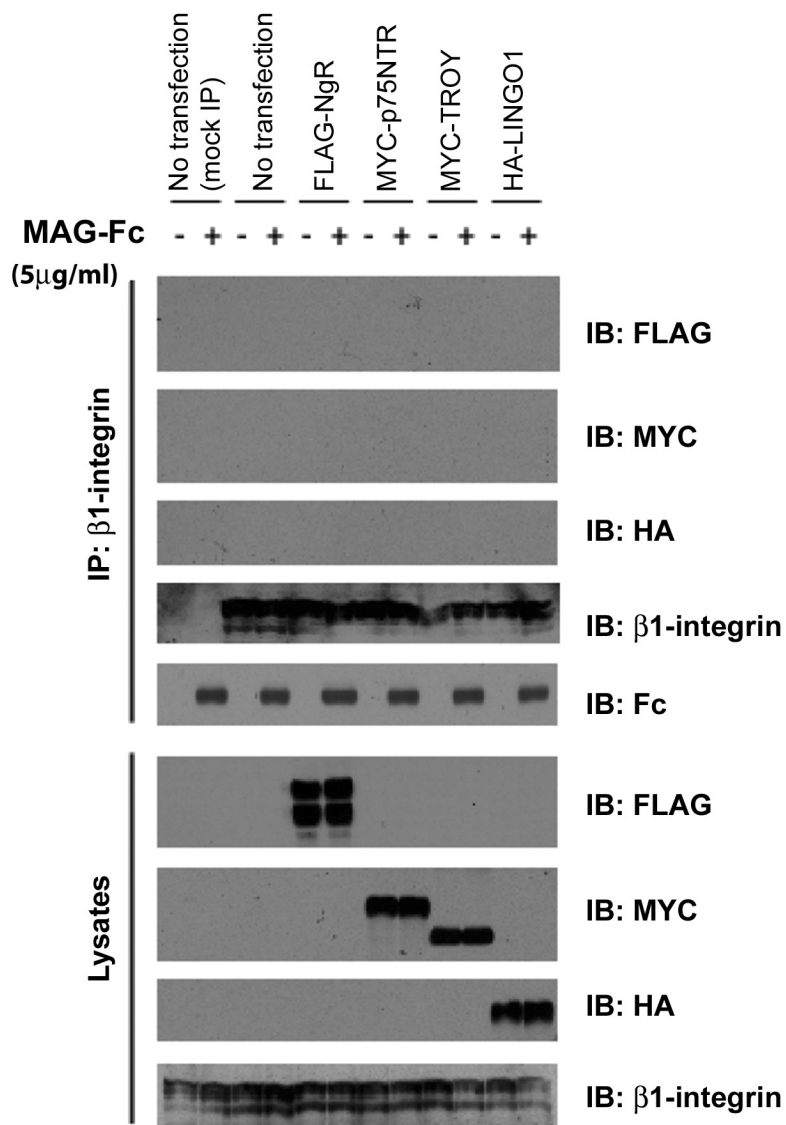

Supplement: Additional file 4 — Lack of interaction between β1-integrin and members of the known NgR receptor complex. Lysates of HEK293 cells transfected with expression constructs for NgR, p75, TROY, or Lingo-1, with or without MAG treatment (5 μg/ml), were immunoprecipitated with anti-β1-integrin antibodies and immunoblotted for the respective components of the NgR receptor complex. Also shown are immunoblots for total cell lysates showing the expression of endogenous β1-integrin and transfected proteins, and reblots for Fc showing a strong association of MAG and β1-integrin in 293 cells. [file 1756-6606-1-10-S4.pdf]

**A**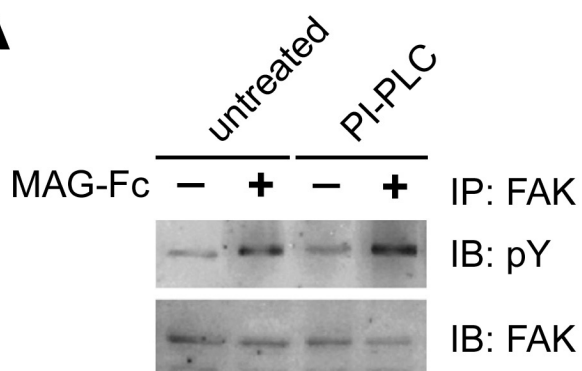**B**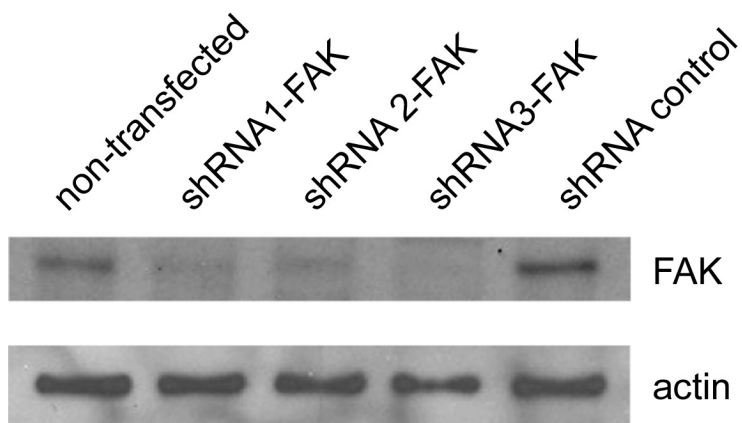

Supplement: Additional file 5 — FAK activation independent of PI-PLC. A. The PI-PLC treatment does not affect MAG-induced phosphorylation of FAK in hippocampal neurons. Hippocampal neurons were treated with saline or PI-PLC (1 units/ml) for 30 min at 37°C, then stimulated with MAG (1 μg/ml) for 15 min. Cell lysates were immunoprecipitated with anti-FAK antibodies and immunoblotted with pY-20 antibody for phosphorylated tyrosine residues. The membrane was reblotted for FAK to show similar loading. B. Knockdown of the expression of endogenous FAK by specific shRNAs. Sample western blot of 3T3 cells were transfected with vectors expressing control shRNA or specific shRNAs against FAK, and then immunoblotted for FAK and β-actin. [file 1756-6606-1-10-S5.pdf]
